# Enhancement of Acid Protease Activity of *Aspergillus oryzae* Using Atmospheric and Room Temperature Plasma

**DOI:** 10.3389/fmicb.2020.01418

**Published:** 2020-06-26

**Authors:** Liang Shu, Xiaoguang Si, Xinda Yang, Wenyan Ma, Jinglan Sun, Jian Zhang, Xianli Xue, Depei Wang, Qiang Gao

**Affiliations:** ^1^Key Laboratory of Industrial Microbiology and Engineering Research Center of Food Biotechnology, Ministry of Education, College of Biotechnology, Tianjin University of Science and Technology, Tianjin, China; ^2^The Institute of Seawater Desalination and Multipurpose Utilization, Ministry of Natural Resources, Tianjin, China; ^3^Tianjin Key Laboratory of Industrial Fermentation Microbiology, Tianjin, China; ^4^Tianjin Engineering Research Center of Microbial Metabolism and Fermentation Process Control, Tianjin, China

**Keywords:** atmospheric and room temperature plasma, *Aspergillus oryzae*, protease, mutation, breeding, fermentation

## Abstract

Atmospheric and room temperature plasma (ARTP) system is a novel and efficient mutagenesis protocol for microbial breeding. In this study, ARTP was employed to treat spores of *Aspergillus oryzae* strain 3.042 for selection of high acid protease producers. With an irradiation time of 150 s at the lethal rate of 90%, 19 mutants with higher acid protease activity were initially selected based on different mutant colony morphology and ratio of the clarification halo of protease activity to the colony diameter. Measurements of the acid protease activity revealed that mutant strain B-2 is characterized by a steady hereditary stability with increased acid protease, neutral protease and total protease activities of 54.7, 17.3, and 8.5%, respectively, and decreased alkaline protease activity of 8.1%. In summary, the identified mutant strain B-2 exhibits great potential for the enhancement of the insufficient acid protease activity during the middle and later stages of soy sauce fermentation.

## Introduction

Soy sauce, a traditional condiment in China, Japan, South Korea, and Southeast Asia, is fermented from soybean and wheat flour and is now used in many countries worldwide. Recent studies have indicated that soy sauce demonstrates many health functions, such as antibacterial, antihypertension, and antioxidant properties, increases in appetite, and the promotion of gastric juice secretion (Kataoka, 2005; Kobayashi, 2005; Chen et al., 2012; Katayama et al., 2017).

Compared with yeasts and lactic acid bacteria, *Aspergillus oryzae* plays a decisive role specifically in the production of soy sauce koji, which determines the utilization of raw proteins and the quality of soy sauce (Luh, 1995; Xu et al., 2013; Zhao et al., 2015a, 2018; Harada et al., 2017). In addition, this organism is also widely used for the manufacturing of various traditionally fermented foods (Zhao et al., 2013; Kitamato, 2015). *A. oryzae* is normally considered GRAS (generally regarded as safe) due to the absence of the aflatoxin gene cluster (Barbesgaard et al., 1992; Machida et al., 2005; Kitamato, 2015; Zhao et al., 2015b). Moreover, it grows rapidly, exhibits a high capability to produce spores, and secretes vast amounts of various hydrolytic enzymes, such as proteases, amylases, glucoamylases, celluloses, and phytases (Sandhya et al., 2005; Kim and Lee, 2008; Liang et al., 2009; Xu et al., 2011; Chen et al., 2012; Kitamato, 2015), as well as organic acids (Ding et al., 2018; Feng et al., 2019). For decades, *A. oryzae* strain 3.042 has been widely adopted by the fermented soy sauce industry in China (Zhao et al., 2012, 2013, 2015a,b; Kitamato, 2015). However, its major proteases are neutral and alkaline proteases, and its limited acid proteases are insufficient for the hydrolysis of the proteins in wheat and soybean when the matrix pH decreases and becomes acidic during the middle and later stages of fermentation (Yang and Lin, 1998; Sandhya et al., 2005; te Biesebeke et al., 2005; Chutmanop et al., 2008; Xu et al., 2011; Zhao et al., 2012). Thus, the proteinaceous raw materials are not sufficiently utilized. Several mutation attempts demonstrate the feasibility of breeding strains with high acid protease activity for the soy sauce fermentation industry (te Biesebeke et al., 2005; Xu et al., 2011; Zhao et al., 2012). Although genetic engineering technology has made great achievements in strain breeding using advanced molecular genetic techniques, the safety of the generated strains is still questioned due to the introduction of foreign genes (Szyjka et al., 2017).

The atmospheric and room temperature plasma (ARTP) system is a novel approach for the mutagenesis of microbial genomes, and it consists of a power supply, a plasma generator, an automatic controller, a water cooler, and a gas supply subsystem (Li et al., 2008; Wang et al., 2010; Winter et al., 2015; Niu et al., 2018; Qiu et al., 2019). The plasma jet generated by ARTP is controlled by the discharged power and gas flow, and its composition mainly includes excited helium atoms, excited oxygen atoms, excited hydrogen atoms, and free •OH radicals (Zhang et al., 2014; Winter et al., 2015; Zhang et al., 2015; Yu et al., 2019). Due to its low voltage radio-frequency power source, low temperature plasma, equal uniform plasma jet, easy operation, and high safety, ARTP has become increasingly popular among microbial breeding operators for its extensive applications (Wang et al., 2010; Zhang et al., 2014; Yang et al., 2019; Zhou et al., 2019). Li et al. (2008) have identified the mechanism of radio-frequency and atmospheric-pressure glow discharges (APGD) directly acted on plasmid DNA and oligonucleotides. In addition, APGD was successfully used for the breeding of *Streptomyces avermitilis* to enhance the production of avermectins (Wang et al., 2010). To date, ARTP, which was developed based on the concept of APGD, has become an efficient breeding method in the field of industrial microbiology (Zhang et al., 2014, 2015; Wu et al., 2015; Zhu et al., 2019).

In the current study, the ARTP mutation system was utilized for the first time to mutate the spores of *A. oryzae* strain 3.042, and some of the mutants with the highest acid protease activity in solid-state fermentation were selected according to the morphology of the mutant colonies and the ratio of the clarification halo which was formed by the milk protein hydrolysis of acid protease to the colony diameter. In addition, the genetic stability of the mutants was also assayed.

## Materials and Methods

### Strain, Reagents, and Culture Media

The *A. oryzae* strain 3.042 was purchased from China General Microbial Culture Collection Center (CGMCC No. 3.00951, Beijing, China) and used as the original strain for ARTP mutagenesis. Strain B-2 (CGMCC No. 8199) was a mutant strain with the supposedly highest acid protease activity by ARTP treatment of *A. oryzae* strain 3.042 in this study.

The bran and bean pulp were kindly supplied by Tianjin Tianli Mature Vinegar Co., Ltd. (Tianjin, China). Casein and Folin-Ciocalteu’s phenol reagent were purchased from Beijing Solarbio Science and Technology Co., Ltd. (Beijing, China). All other reagents were of analytical grade.

Potato dextrose agar (PDA) medium was used for the conventional culture and subculture of *A. oryzae* strain 3.042 and its mutants (Zhao et al., 2012). The screening medium was composed of 1% non-fat dry milk and 1.5% agar, which was sterilized at 115°C for 20 min before mixing, and the medium was adjusted to pH 3.0 using HCl under sterile conditions. The solid-state fermentation medium consisted of bean pulp, bran, and tap water at a ratio of 6:4:8 and was sterilized at 121°C for 30 min.

### Solid-State Fermentation

Spores of *A. oryzae* strain 3.042 or its mutants were obtained from a 3-day PDA slant culture, washed with 10 mL of sterile normal saline, transferred into a 250-mL conical flask with 10 glass beads, dispersed at 200 r/min and 30°C for 1 h, and filtered using sterilized paper. The prepared spore suspension was then diluted to approximately 1 × 10^6^ CFU/mL for ARTP mutagenesis. For the solid-state fermentation, 2 mL of the spore suspension was added to a 500-mL conical flask with 63 g of solid fermentation medium, and the mixture was cultured at 30°C for 48 h. The flasks were shaken every 6 h to disperse the solid medium.

### ARTP Mutagenesis

The ARTP system [model ARTP-II, Beijing Si Tsingyuan (BEST) Biotechnology Co., Ltd., Beijing, China] was utilized to mutate the spores of *A. oryzae* strain 3.042 in this study.

A 10-μL spore suspension was spread over an iron dish and then air-dried under aseptic conditions. The mutation conditions were as follows: 120 W of the radio-frequency power input, 10 L/min helium gas flow rate, 2 mm distance between the plasma jet nozzle and iron dish, but only an irradiation time of 30–240 s at 30 s intervals under the plasma jet as the variable quantity. The untreated samples served as the control of 100% survival rate.

### Pre-screening and Re-screening Processes

For the pre-screening process, after exposure for an optimal time, the iron dish was placed in a 1.5-mL sterile Eppendorf tube and cells were eluted using sterile normal saline. A 100-μL mutated spore suspension was respectively spread on a screening agar plate for the ratio of clarification halo to the colony diameter (namely, zone/colony ratio) and a PDA plate for morphology observation for 2–3 days incubation at 37°C. Colonies with either an apparently clarification halo due to protease action or varied colony morphology were isolated (Wang et al., 2010) and activated by culturing on PDA plates for 2–3 days at 37°C. The activated mutants were selected and then inoculated on screening agar plate to measure the clarification halo and colony diameter after growth of 2–3 days at 37°C.

The survival rate of the spores at different ARTP irradiation times of 30–240 s was determined in triplicate and calculated using the following equation:

Survival⁢rate=(S/U)×100%

where *S* is the total CFU of the mutants and *U* is the total CFU of the original strain without ARTP treatment on the PDA agar plate.

In the re-screening process, the mutants identified from the pre-screening process were cultured through solid-state fermentation, and the individual acid protease activity was measured as described below.

### Protease Activity Assay

The protease activity was determined in triplicate using the Folin–Ciocalteu’s phenol reagent method (Lowry et al., 1951; General Administration of Quality Supervision, 2012; Zhang et al., 2019). The crude protease extract was prepared as follows: 5 g of solid-state fermentation medium was mixed with 100 mL of the appropriate buffer. In particular, lactic acid buffer (0.05 mol/L, pH 3.0), phosphate buffer (0.02 mol/L, pH 7.2) and sodium tetraborate-NaOH buffer (0.0125 mol/L sodium tetraborate, 0.043 mol/L NaOH, pH 10.0) were used for the crude extraction of acid, neutral, and alkaline protease activity, respectively. Samples were shaken for 1 h at 180 r/min and 40°C. Next, mixtures were centrifuged at 10,000 × *g* for 10 min at 4°C and the protease activities in the supernatants were determined in triplicate. One unit of acid protease was defined as the amount of enzyme that produces 1 μg of tyrosine per min under the assay conditions. The measured protease activities were calculated as the mean ± SD values of three independent experiments.

## Results

### Survival Rate of *A. oryzae* 3.042 Spores After ARTP Treatment

Previous studies (Wang et al., 2010; Zhang et al., 2014; Winter et al., 2015) revealed the variable operating parameters for ARTP, including the radio frequency power, gas flow rate, distance between the plasma generator and sample plate, the type of plasma working gas, and the irradiation treatment time. In the current study, the irradiation time was the unique variable factor, and the other parameters were fixed.

**FIGURE 1:**
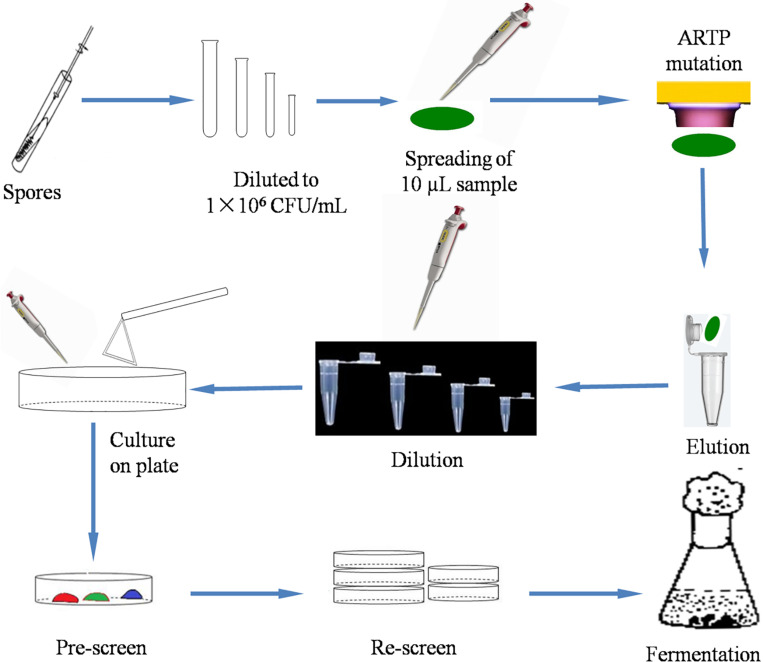
Schematic flow diagram of the mutation of *A. oryzae* strain 3.042 using ARTP.

**FIGURE 2:**
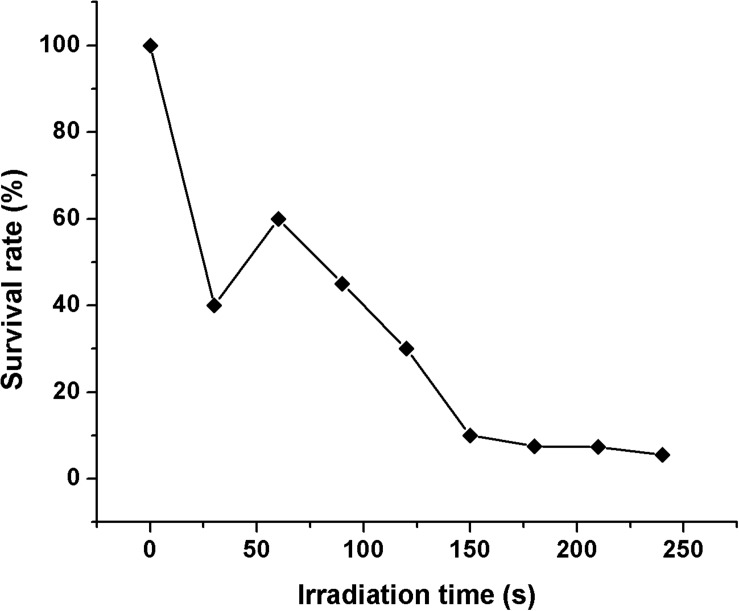
The survival rate curve of *A. oryzae* strain 3.042 according to the ARTP irradiation time.

In this study, a schematic flow diagram of the ARTP mutation experiment is shown in Figure 1. The survival curve for *A. oryzae* spores irradiated by ARTP is a “saddle-shaped” curve (Figure 2). The survival rate of the ARTP-treated *A. oryzae* 3.042 spores sharply decreased with an increase in the ARTP irradiation time; however, after the saddle point at 60 s, the survival rate of the spores decreased to 30.1, 10.0, and 7.5%, after irradiation using the ARTP plasma jet for 120, 150, and 180 s, correspondingly. When the spores were treated for 240 s under the same conditions, nearly none of the spores survived. The high lethal rate combined with a highly positive mutation rate is desirable for the efficient mutagenesis and screening of mutants. Thus, an irradiation time of 150 s was selected as the optimal exposure time to obtain a suitable survival rate for the efficient mutagenesis and screening in this study.

### Variation of Colony Morphology After ARTP Treatment

**FIGURE 3:**
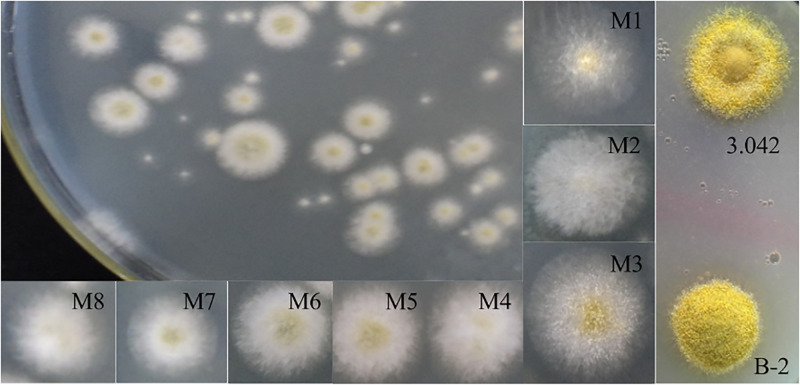
Variation in the colony morphologies of the ARTP-treated spores. Mutants M1–M8 were cultured on PDA plates at 37°C for 2 days, whereas the wild-type 3.042 and mutant B-2 strains were cultured on a screening agar plate at 37°C for 5 days.

The colony morphology of many mutant spores on PDA plates differed from that of strain 3.042 due to the effect of ARTP treatment. As shown in Figure 3, the mutant colonies demonstrated different appearances after ARTP treatment, e.g., mutant M1 showed less dense mycelia and more abundant spores compared to M2. However, M3 shared characteristics with both M1 and M2 because it had both abundant spores and sufficient mycelia. M4 and M5 were adjacent colonies, but their appearances were clearly different. In addition, M6 demonstrated the colony morphology of convex. The colony morphology of M7 was with a neat edge and umbonate, while M8 presented an irregular edge and a flocculent colony morphology.

Although there were not visible differences between the mycelial morphology under the microscope, the colony morphology of mutant strain B-2 and *A. oryzae* strain 3.042 were significantly different on the acidic screening agar plate (Figure 3), such difference might be caused by the altered protease activity at acidic pH.

### Screening Procedure

In the pre-screening stage, the morphology of the colony and the ratio of the clarification halo by protease activity to the colony diameter were the predominant factors used for selection. The morphology of the mutants might change to be diverse due to mutations in the genome. However, previous studies indicated that the phenotype of the microorganisms was the common combination of genotype and environment (Wang et al., 2010), and mutations in genome could not always indicate an improvement of acid protease activity.

**FIGURE 4:**
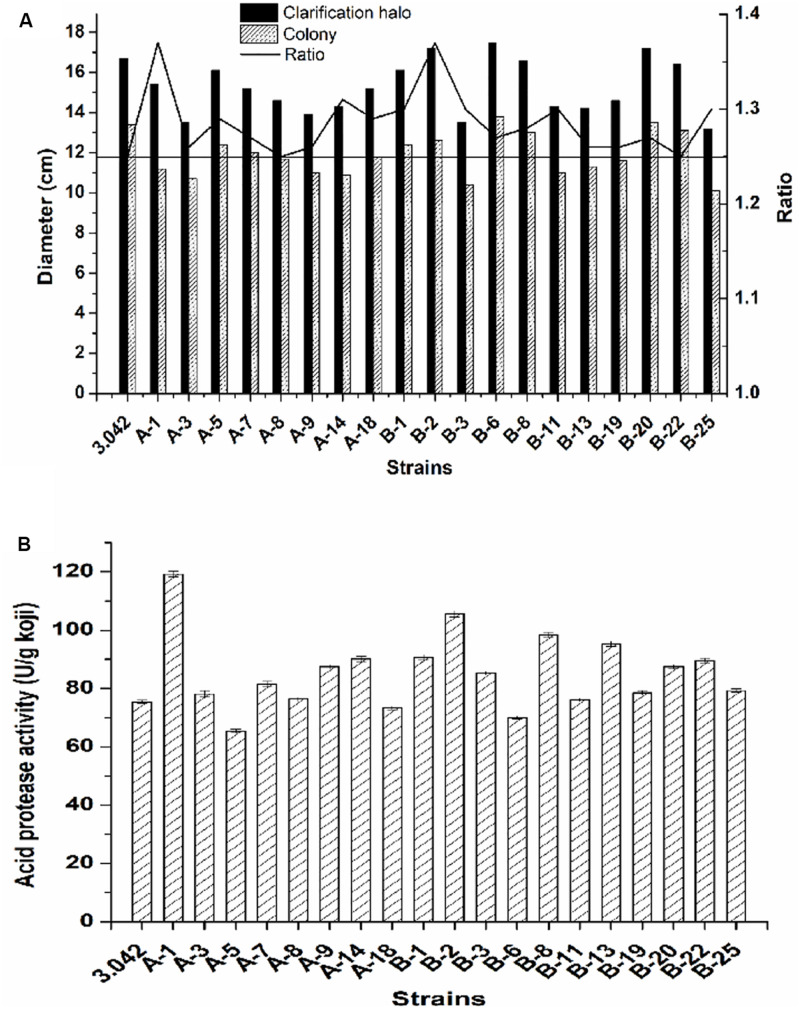
Screening strategy for high acid protease activity mutant. **(A)** Ratio of the clarification halo to the colony diameter. **(B)** Acid protease activity of the 19 mutants and the original strain 3.042.

Here, a total number of 75 colonies with either altered morphology or big clarification halo were first picked out as the potential targeted mutants based on their altered morphology or high zone/colony ratio and cultivated on PDA plates for 2–3 days at 37°C. Subsequently, 19 of potential mutants with higher zone/colony ratio were selected for further re-screening (Figure 4A).

As previously mentioned, the pre-screening did not provide sufficient evidence to determine the activity of acid protease. To obtain the mutant with the best potential for practical applications, the re-screening procedure was necessary to select the mutant with the highest acid protease activity. The acid protease activities of the selected 19 mutants were assayed by the re-screening process (Figure 4B). As shown in Figure 4, there was no linearity between enzyme activity and the ratio of clarification halo to colony diameter, the positive mutation rate of acid protease activity is 63.2% while the negative mutation rate is 10.5%, where the positive/negative mutation rate is defined as acid protease activity beyond 5% deviation of that of the control.

### Genetic Stability of the Examined Mutants

**FIGURE 5:**
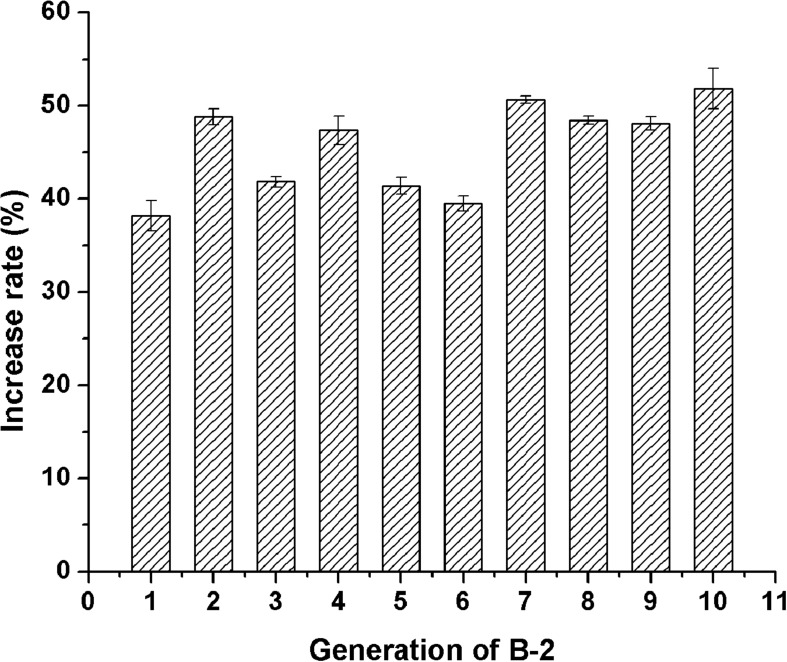
Stability of the acid protease activity of B-2 after 10-generation subcultures.

Of the 19 examined strains, 5 mutants, namely A-1, B-1, B-2, B-8, and B-13, exhibited much higher acid protease activities compared to the original strain 3.042 by increases of 58.1, 20.3, 39.9, 30.4, and 26.4%, respectively (Figure 4B). However, at the 10th-generation subculture, only mutant B-2 was the only mutant to maintain a highest acid protease activity of 114.6 U/g koji (Figure 5). Thus, mutant B-2 was selected for the study of its genetic stability in a solid-state fermentation process.

### Total Protease Activity of Mutant B-2

**FIGURE 6:**
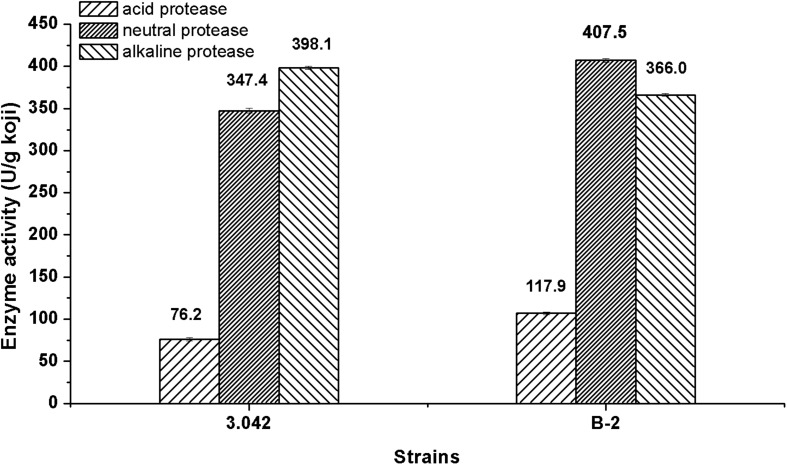
Total protease activity of the mutant B-2 and the original strain 3.042.

The overall performance of *A. oryzae* strain in enzymes activity is desired for soy sauce production (Xu et al., 2013). The current study demonstrated that the mutant strain B-2 exhibited an overall 8.5% increase in total acid, neutral and alkaline protease activity compared with *A. oryzae* strain 3.042. Furthermore, the strain B-2 exhibited increases of 54.7 and 17.3% in acid protease and neutral protease activity, respectively, whereas the activity of alkaline protease showed a decrease of 8.1% as compared with the original strain 3.042 (Figure 6).

## Discussion

The fermentation strain is a key factor for soy sauce production. Mutation breeding is one of the most conventional and effective methods to improve microbial performance. However, many microbes were reported to express a phenomenon of resistant saturation when repeatedly mutated by the same mutagen (Wang et al., 2007). Originally, *A. oryzae* strain 3.042 was obtained from a wild-type strain via multiple UV mutation, and the acid protease activity improvement has become more and more difficult. That may be due to the resistant saturation of *A. oryzae* strain 3.042 (Kim and Lee, 2008; Wang et al., 2007). Therefore, a novel mutation protocol, rather than UV treatment, is expected here to treat strain 3.042 with good outcomes. The ARTP plasma jet has been used for strain breeding in recent years and exhibits a great effect owing to its convenient operation compared to space mutagenesis or molecular breeding (Wang et al., 2010; Zhang et al., 2014; Winter et al., 2015). Compared to the “traditional” techniques, ARTP can generate diversified damage mechanism to genetic material, which resulting in the diversity of mutant types. This property puts ARTP possess unique advantages and higher positive mutation rates than UV mutagenesis in mutation breeding, especially in the microorganism which has complex metabolic networks (Wang et al., 2010; Zhang et al., 2014). It has been shown that the chemically active species instead of UV, intense electric field, heat and charged particles mainly break the double chains of DNA during ARTP treatment (Li et al., 2008). The effect of plasma on the microorganism is dependent on the operating conditions, such as power input, treatment distance, gas flow rate and treatment time. The only requirement for the usage of ARTP system is to prepare the spore or microbial cell suspension before mutation operation at room environment (Wang et al., 2010; Zhang et al., 2014).

In this study, we demonstrated that the ARTP mutation system is an efficient protocol for the mutagenesis and mutant selection of soy sauce producing *A. oryzae* strain 3.042 with highly improved acid protease activity. The survival curve by the ARTP plasma jet treatment is perfectly consistent with the “saddle-shape” curves obtained using low-energy heavy ion implantations (Han et al., 2009). In addition, the saddle point appeared at an irradiation time of 60 s. The potential interpretation might include several complicated factors, one reason is the combined damage effects of energy and mass deposition, momentum transfer and electric charge exchange of the ARTP plasma jet, another is the DNA repair mechanism involved in the targeted microorganism (Meng, 2001).

After ARTP treatment, the isolation and screening of target mutants are key procedures in microbial breeding. To screen productive protease-secreting microorganisms more efficiently, the adopted method must follow these principles: (a) the clarification halo of protease activity should be very large compared with that of the original strain and (b) the scale of the microbial colony should be sufficiently massive to ensure the presence of biomass in the fermentation process. The detection of acid protease-making microorganisms is based on the theory that acid proteases hydrolyze the opaque milk protein in the screening medium to a clarification halo in an acidic environment. Due to the large amount of samples in the breeding stage, a two-step strategy of pre-screening and re-screening procedures was applied for effective mutant selection in the current study.

Changes in the colony morphology indicated the modification of few genes in the genome. However, it remains unclear whether there is an essential relationship between acid protease activity and colony morphology.

In mutation breeding, genetic stability of a microbial mutant is vital for the fermentation process. Although the protease yield may be improved at its early generations due to the treatment of mutagens, the degeneration of performance often occurs to the industrial mutants due to the back mutations. Therefore, it is a routine work to determine the genetic stability of the mutant strain for the rejuvenation to eliminate the degenerated descendent cells during subculture processes (Meng, 2001). A common method to avoid unstable protease overproduction or degeneration is to breed for the mutant strains and then assay the stability of the protease activity in subcultures. By this way, a mutant strain B-2, which maintained a steady increased total protease activity compared with that of strain 3.042, is selected, and expected to effectively surpass the disadvantages associated with strain 3.042 during the middle and later fermentation stages of soy sauce production.

## Conclusion

In this study, we used the ARTP mutation system to mutagenize soy sauce producing *A. oryzae* strain 3.042 and finally got a mutant strain B-2 with a steady hereditary stability after using an optimal solution for 150 s of radiation time and 90% of the fatality rate. Its acid protease, neutral protease, and total protease activities increased by 54.7, 17.3, and 8.5%, respectively, and alkaline protease activity decreased by 8.1% compared to the original strain. Thus, the ARTP mutation system is very useful in selecting a strain with excellent traits.

## Data Availability Statement

All datasets presented in this study are included in the article/supplementary material.

## Author Contributions

LS and XS performed the experiments and analyzed the data. XY helped to conduct the ARTP mutagenesis with WM and JS. JZ, XX, and DW corrected the manuscript. QG supervised the project. All authors contributed to the article and approved the submitted version.

## Conflict of Interest

The authors declare that the research was conducted in the absence of any commercial or financial relationships that could be construed as a potential conflict of interest.
